# An epidemiological study of the predictors of multidrug resistance and methicillin resistance among *Staphylococcus* spp. isolated from canine specimens submitted to a diagnostic laboratory in Tennessee, USA

**DOI:** 10.7717/peerj.15012

**Published:** 2023-03-24

**Authors:** Jennifer Lord, Nick Millis, Rebekah Duckett Jones, Brian Johnson, Stephen A. Kania, Agricola Odoi

**Affiliations:** Biomedical and Diagnostic Sciences, University of Tennessee, Knoxville, TN, United States of America

**Keywords:** Multidrug resistance, Methicillin resistance, Staphylococcus, Epidemiology, Canine, Generalized estimating equations, GEE

## Abstract

**Background:**

Understanding drivers of multidrug resistance (MDR) and methicillin resistance, which have increased among canine staphylococcal isolates, is essential for guiding antimicrobial use practices. Therefore, the objective of this study was to identify predictors of MDR and methicillin resistance among *Staphylococcus* spp. commonly isolated from canine clinical specimens.

**Methods:**

This retrospective study used records of canine specimens submitted to the University of Tennessee College of Veterinary Medicine Clinical Bacteriology Laboratory for bacterial culture and antimicrobial susceptibility testing between 2006 and 2017. Records from 7,805 specimens positive for the following *Staphylococcus* species were included for analysis: *Staphylococcus pseudintermedius, Staphylococcus aureus, Staphylococcus coagulans* (formerly *Staphylococcus schleiferi* subspecies *coagulans*), and *Staphylococcus schleiferi* (formerly *S. schleiferi* subsp. *schleiferi*). Generalized linear regression models were fit using generalized estimating equations (GEE) to identify predictors of MDR (defined as resistance to three or more antimicrobial classes) and methicillin resistance among these isolates.

**Results:**

Multidrug resistance (42.1%) and methicillin resistance (31.8%) were relatively common. Isolates from skeletal (joint and bone) specimens had the highest levels of MDR (51.3%) and methicillin resistance (43.6%), followed by cutaneous specimens (45.8% multidrug-resistant, 37.1% methicillin resistant). *Staphylococcus* species, specimen site, and clinical setting were significant (*p* < 0.01) predictors of both outcomes. Compared to *S. pseudintermedius, S. schleiferi* had higher odds of methicillin resistance, while *S. coagulans* and *S. schleiferi* had lower odds of MDR. The odds of both MDR and methicillin resistance for isolates from hospital patient specimens were significantly higher than those from referral patients for urine/bladder and otic specimens. Odds of MDR among isolates from skeletal specimens of hospital patients were also higher than those of referral patients.

**Conclusions:**

*Staphylococcus* isolates in this study had substantial levels of MDR and methicillin resistance. Differences in the odds of these outcomes between referral and hospital patient isolates did not persist for all specimen sites, which may reflect differences in diagnostic testing and antimicrobial use practices with respect to body site or system. Judicious antimicrobial use, informed by culture and susceptibility testing, is important to limit treatment failures and curb selection pressure.

## Introduction

Multidrug resistance (MDR) and methicillin resistance have been reported with increasing frequency among canine staphylococcal isolates in recent decades (Morris et al., 2017). Methicillin-resistant organisms are resistant to all antibiotics in the *β*-lactam class with the exception of some newer cephalosporins (Clinical and Laboratory Standards Institute, 2018), while multidrug resistance is defined as resistance to at least one agent in three or more antimicrobial classes (Magiorakos et al., 2012; Sweeney et al., 2018). Therefore, few antimicrobial agents are effective for treatment of infections with these organisms, particularly staphylococci that are both multidrug- and methicillin-resistant. Moreover, even fewer antimicrobial agents are available for use in veterinary patients compared to human patients, exacerbating the clinical challenge of treating resistant staphylococcal infections in companion animals. The colonization and infection of domestic dogs with these resistant organisms also presents a public health concern because of the close contact between humans and canine companions in many households (Pomba et al., 2017).

Methicillin-resistant *Staphylococcus pseudintermedius* (MRSP) and *Staphylococcus aureus* (MRSA) are considered to be among the most important antimicrobial resistance threats to human health presented by companion animals, since these organisms can be transmitted directly from animals to humans (Pomba et al., 2017). *S. pseudintermedius,* which is the most common commensal of canine skin and mucosa but is not part of the normal human flora (Bannoehr & Guardabassi, 2012), can cause zoonotic infections, which may be invasive and can occur in individuals without underlying immune dysfunction (Stegmann et al., 2010; Somayaji et al., 2016; Kmieciak & Szewczyk, 2018; Ference et al., 2019; Blondeau et al., 2020). Dog ownership is a risk factor for *S. pseudintermedius* infection in humans (Ference et al., 2019), and carriage of *S. pseudintermedius* is more common among members of the veterinary profession compared to the general population (Morris et al., 2010; Paul et al., 2011). *S. aureus* is a well-known human commensal and opportunistic pathogen that is frequently implicated in nosocomial outbreaks (Tong et al., 2015). Canine carriage and infection with *S. aureus* are thought to occur mainly through contact with colonized humans, but bi-directional transmission can occur within households (Van Duijkeren et al., 2004; Rutland et al., 2009; Morris et al., 2012; Sasaki et al., 2012; Mork et al., 2020).

Two other clinically important staphylococci of domestic dogs are *Staphylococcus coagulans* and *Staphylococcus schleiferi*. Formerly considered subspecies of *S. schleiferi* (*S. schleiferi* subsp. *coagulans* and *S. schleiferi* subsp. *schleiferi*), recent research has supported their reclassification into two distinct species (Madhaiyan, Wirth & Saravanan, 2020). Both organisms are considered commensals of canine skin that may act as opportunistic pathogens, and have primarily been implicated in infections such as pyoderma and otitis externa (Cain, Morris & Rankin, 2011; Frank et al., 2003; Kania et al., 2004; Kunder et al., 2015; Lee et al., 2019; May, Kinyon & Noxon, 2012). As with other coagulase-negative *Staphylococcus* species, methicillin resistance tends to be relatively common among *S. schleiferi* isolates (Becker, Heilmann & Peters, 2014; Cain, Morris & Rankin, 2011; Frank et al., 2003; Jones et al., 2007; Kania et al., 2004; Kunder et al., 2015). *S. schleiferi* and *S. coagulans* infections in human patients have been described in numerous case reports, including several infections presumed to be the result of zoonotic *S. coagulans* transmission from pet dogs (Ezaki et al., 2020; Hernández et al., 2001; Kumar et al., 2007; Swe et al., 2016; Thibodeau et al., 2012; Tzamalis et al., 2013; Yarbrough et al., 2017).

Recent analysis of antimicrobial susceptibility data from canine specimens submitted to the Clinical Bacteriology and Mycology Laboratory at the University of Tennessee College of Veterinary Medicine (UTCVM) identified temporal increases in the percentage of MRSA and MRSP isolates, as well as evidence of emerging chloramphenicol resistance among *S. pseudintermedius* isolates (Lord et al., 2022). These concerning findings warrant further exploration to identify drivers of MDR and methicillin resistance among canine staphylococci in the region. While previous research has investigated predictors of these outcomes, the factors associated with MDR and methicillin resistance have varied between studies (Soares Magalhães et al., 2010; Cain, Morris & Rankin, 2011; Huerta et al., 2011; Weese et al., 2012; Eckholm et al., 2013; Gandolfi-Decristophoris et al., 2013; Lehner et al., 2014; Zur, Gurevich & Elad, 2016; Conner et al., 2018; Ortiz-Díez et al., 2020), which may reflect differences in study populations as well as the local epidemiology of resistant staphylococci in different geographic locations. Identifying drivers of MDR and methicillin resistance among canine staphylococci is essential to improve our understanding of the trends observed in this region, and to inform veterinary efforts to respond to the emerging challenge. This information can be disseminated to practitioners in the study area to guide antimicrobial use practices. Furthermore, in light of the potential human health implications of zoonotic transmission and transfer of antimicrobial resistance genes from canine staphylococci (Pomba et al., 2017), identifying these drivers is essential for informing the development of One Health strategies to prevent or mitigate threats to human and animal health. The One Health framework recognizes the importance of interdisciplinary collaboration to promote the health of humans, animals, and the environment (One Health High-Level Expert Panel (OHHLEP) et al., 2022). Therefore, the objective of this study was to identify predictors of multidrug resistance and methicillin resistance among *Staphylococcus* spp. commonly isolated from canine clinical specimens submitted to the Clinical Bacteriology and Mycology Laboratory at the University of Tennessee College of Veterinary Medicine between 2006 and 2017.

## Methods

### Ethics approval and consent for publication

This retrospective study involved analysis of existing bacteriology diagnostic laboratory data and corresponding hospital records obtained as part of routine clinical care from client-owned animals. At the time of collection, all owners provide consent that upon completion of diagnostic testing, excess biological specimens may be used for teaching or research purposes. The University of Tennessee Institutional Animal Care and Use Committee (IACUC) concluded that the study did not use animals or animal tissues and therefore did not require IACUC oversight.

### Data source

Hospital and laboratory records for this retrospective study were obtained from the University of Tennessee College of Veterinary Medicine (UTCVM) Veterinary Teaching Hospital and Clinical Bacteriology Laboratory. Records for unique canine clinical specimens submitted for bacterial culture and susceptibility testing between 2006 and 2017 that were positive for *S. pseudintermedius, S. aureus, S. coagulans* (formerly *S. schleiferi* subsp. *coagulans*) or *S. schleiferi* (formerly *S. schleiferi* subsp. *schleiferi*) were included in the analysis. Data extracted from these records included patient identification and medical record numbers, age, sex, breed, collection site, *Staphylococcus* species, antimicrobial susceptibility and *mecA* polymerase chain reaction (PCR) assay results, and patient type (hospital patient specimen *vs.* submission by referring veterinarian).

### Antimicrobial susceptibility testing and *mecA* PCR

Bacterial isolation and antimicrobial susceptibility testing of *Staphylococcus* spp. isolates were performed as previously described (Jones et al., 2007). Isolates obtained *via* aerobic bacterial culture were identified with a biochemical identification process, using a tube coagulase test, fermentation tests using phenol red broth with lactose and with trehalose, and the Voges-Proskauer test (Jorgensen, Carroll & Pfaller, 2015). All canine *Staphylococcus intermedius* group (SIG) isolates were reported as *S. pseudintermedius* (Sasaki et al., 2007; Devriese et al., 2009; Vrbovská et al., 2020).

Antimicrobial susceptibility testing was performed according to Clinical and Laboratory Standards Institute (CLSI) guidelines (Clinical and Laboratory Standards Institute, 2006; Clinical and Laboratory Standards Institute, 2008; Clinical and Laboratory Standards Institute, 2010; Clinical and Laboratory Standards Institute, 2012; Clinical and Laboratory Standards Institute, 2018). The standard antimicrobial susceptibility panel included ampicillin, amoxicillin/clavulanic acid, cefoxitin, cefpodoxime, cephalothin, chloramphenicol, clindamycin, erythromycin, marbofloxacin, oxacillin, penicillin, tetracycline, and trimethoprim/sulfamethoxazole (TMS). Isolates resistant to multiple drug classes were also tested for susceptibility to amikacin, doxycycline, minocycline, and rifampin. All cutaneous isolates were also tested for susceptibility to amikacin, while otic and urinary isolates were tested for susceptibility to gentamicin. Antimicrobial susceptibility for the majority of isolates included in this study was determined using disk diffusion testing on cation-adjusted Mueller-Hinton agar, but some (approximately 5% of the isolates) were tested with a microbroth dilution method using an automated susceptibility testing system (BioMerieux, n.d.; Jones et al., 2007). In addition, ciprofloxacin and enrofloxacin were included in a minimum inhibitory concentration (MIC) panel used for some isolates.

Oxacillin breakpoints used for *S. pseudintermedius, S. coagulans* and *S. schleiferi* were ≤17 mm (resistant) and ≥18 mm (susceptible) (Bemis et al., 2009; Schissler et al., 2009). Otherwise, interpretive breakpoints from CLSI guidelines in place during the year of submission were followed (Clinical and Laboratory Standards Institute, 2006; Clinical and Laboratory Standards Institute, 2008; Clinical and Laboratory Standards Institute, 2010; Clinical and Laboratory Standards Institute, 2012; Clinical and Laboratory Standards Institute, 2018). Conventional PCR was used for *mecA* gene detection during all years of the study except 2006, when real-time PCR was used (Rosato, Craig & Archer, 2003; Jones et al., 2007; Bemis et al., 2009).

### Data management

Data management, statistical analysis and generation of figures were performed using SAS version 9.4 and R software (SAS Institute Inc., 2017; R Core Team, 2020). Results of antimicrobial susceptibility testing and *mecA* PCR assay were excluded if interpretations of repeated tests were inconsistent. Therefore, susceptibility test results for amikacin (two isolates), cefoxitin (two isolates), cefpodoxime (two isolates), and oxacillin (six isolates) were excluded, along with results of *mecA* PCR assay for 14 isolates. In total, *mecA* PCR results for 4,152 *Staphylococcus* spp. isolates were included in the analysis.

Results of antimicrobial susceptibility testing were classified into two groups, susceptible or resistant. Isolates originally classified as “intermediate”, “resistant” or “not susceptible” were included in the “resistant” group (Magiorakos et al., 2012). Isolates were classified as antimicrobial resistant if they were not susceptible to an agent in at least one antimicrobial class, and multidrug-resistant if they were not susceptible to an agent in at least three antimicrobial classes, excluding intrinsic resistance (Magiorakos et al., 2012; Sweeney et al., 2018). Methicillin resistance was determined based on results of disk diffusion susceptibility testing (using cefoxitin for *S. aureus* and oxacillin for all other species), confirmed with positive *mecA* PCR assay (Clinical and Laboratory Standards Institute, 2018).

Specimen collection sites were categorized as skin, ear, urine or bladder, skeletal (joint and bone samples), mucosa (nasal, oral cavity, and vaginal samples), and other. “Other” sites included: bile (two isolates), blood (22), brain (two), catheter (one), cerebrospinal fluid (three), kidney (one), liver (six), lung (two), lymph node (eight), mammary/milk (15), muscle (one), ocular/optic (115), pericardium (two), peritoneum (10), pleura (four), reproductive tract (female) (68), reproductive tract (male) (29), salivary gland (one), semen (two), spleen (one), thorax (one), trachea, tracheal/bronchial or transtracheal wash (37), unspecified abscess (11), unspecified draining tract (one), unspecified respiratory tract (116), unspecified surgical site (10), and unspecified wound (four). Specimen collection site was missing for one isolate. Nine isolates (0.1%) that did not have a single, unambiguous location listed for specimen site were also included in the “other” category.

### Statistical analysis

Since the Kolmogorov–Smirnov test indicated that distribution of the number of antimicrobial classes to which *Staphylococcus* spp. isolates were resistant was non-normal, median and interquartile range (IQR) were used as measures of central tendency and dispersion. Proportions and 95% confidence intervals were computed to investigate the distribution of multidrug resistance and methicillin resistance with respect to clinical characteristics. Generalized linear regression models were fit to the data using generalized estimating equations (GEE), specifying binomial distribution, logit link, and an exchangeable working correlation structure, to identify significant predictors of MDR and methicillin resistance. *Staphylococcus* spp. isolates from the same patient were treated as clustered observations. Since a substantial percentage of records from specimens submitted by referring veterinarians were missing patient signalment information, age, breed, and sex were not included as potential explanatory variables in the modeling process. Instead, the analysis focused on clinical factors including bacterial species, specimen site and patient type. Associations of MDR and methicillin resistance with these potential explanatory variables were first assessed using univariable models. Explanatory variables that exhibited significant univariable associations at a liberal *p*-value of < 0.15 were considered for inclusion in multivariable models. The two multivariable models (MDR and methicillin resistance models) were built using backwards elimination, with a cutoff *p*-value of ≤ 0.05. Explanatory variables were considered confounders if, upon removal, the coefficients for any of the other variables in the model changed by a magnitude of 20% or greater, and retained regardless of statistical significance. Biologically meaningful interaction terms were assessed for significance and retained in the model if their inclusion improved overall model fit. Changes in the value of the modified quasi-likelihood information criterion (QIC_u_) were used to guide variable selection (Hardin & Hilbe, 2012).

## Results

### Descriptive statistics

As many as 7,805 *Staphylococcus* spp. isolates met the inclusion criteria for the study, including 4,689 isolates from canine patients with single specimens. The remaining 3,116 isolates were from 1,220 patients that had multiple specimens submitted for bacterial culture and susceptibility testing during the study period. In total, 6,453 *S. pseudintermedius* isolates, 330 *S. aureus* isolates, 860 *S. coagulans* isolates, and 162 *S. schleiferi* isolates were included for analysis. Together, isolates from cutaneous (4,387) and otic (1,310) specimens comprised nearly three quarters of all isolates in the study (73.0%). Urine and bladder isolates also represented a considerable percentage of *Staphylococcus* spp. isolates in the study (13.3%). Skeletal (joint and bone) and mucosal specimens comprised 5.9% and 1.6% of the isolates in the study, respectively.

The number of distinct antimicrobial classes to which the *Staphylococcus* spp. isolates exhibited resistance ranged from 0 to 8 (median = 2, interquartile range (IQR) = 1 − 5) (Table 1). Among isolates of the four *Staphylococcus* species included in the study, resistance to the *β*-lactam class of antibiotics was the most common (Table 2). Multidrug resistance was exhibited by 42.1% of the isolates, while 31.8% of the *Staphylococcus* spp. isolates were methicillin-resistant. Multidrug resistance was most common among *S. pseudintermedius* (45.5%) and *S. aureus* isolates (40.9%), while *S. schleiferi* had the highest percentage of methicillin-resistant isolates (56.9%) (Table 2). While the majority of methicillin-resistant isolates (83.1%) were also multidrug-resistant, compared to just 21.1% of methicillin-susceptible isolates, the levels of MDR among methicillin-resistant isolates differed by *Staphylococcus* species. For instance, while 90.5% of methicillin-resistant *S. pseudintermedius* (MRSP) and 78.6% of methicillin-resistant *S. aureus* (MRSA) isolates were multidrug-resistant, MDR was less common among methicillin-resistant *S. coagulans* (45.2%) and methicillin-resistant *S. schleiferi* (41.3%) isolates. The most common pattern of resistance among multidrug-resistant *Staphylococcus* spp. isolates included resistance to aminoglycosides, *β*-lactams, fluoroquinolones, folate pathway inhibitors, lincosamides, macrolides, and tetracyclines (784 isolates) (Table 3). This was also the most common pattern among methicillin-resistant *Staphylococcus* spp. isolates, exhibited by 672 methicillin-resistant isolates.

**Table 1:** Distribution of antimicrobial resistance among *Staphylococcus* spp. isolated from canine specimens submitted to a diagnostic laboratory in Tennessee, USA (2006–2017).

**Number of drug classes**			**Percent of isolates (number resistant/total number of isolates)**			
			***S. pseudintermedius***	***S. aureus***	***S. coagulans***	***S. schleiferi***
		0	13.3 (857/6453)	7.3 (24/330)	43.0 (370/860)	22.8 (37/162)
AMR^a^		1	26.9 (1736/6453)	37.3 (123/330)	17.2 (148/860)	22.8 (37/162)
		2	14.4 (927/6453)	14.6 (48/330)	19.8 (170/860)	27.2 (44/162)
	MDR^b^	3	7.1 (455/6453)	12.1 (40/330)	17.1 (147/860)	19.1 (31/162)
		4	4.7 (305/6453)	22.7 (75/330)	2.1 (18/860)	3.1 (5/162)
		5	5.0 (320/6453	5.2 (17/330)	0.8 (7/860)	3.1 (5/162)
		6	13.9 (894/6453)	0.9 (3/330)	0 (0/860)	1.2 (2/162)
		7	13.7 (886/6453)	0 (0/330)	0 (0/860)	0 (0/162)
		8	1.1 (73/6453)	0 (0/330)	0 (0/860)	0.6 (1/162)

**Notes.**

a Antimicrobial resistant.

b Multidrug resistant.

Light gray shading indicates antimicrobial resistance (resistance to one or more antimicrobial classes). Dark gray shading indicates multidrug resistance (resistance to three or more antimicrobial classes).

**Table 2:** Antimicrobial resistance patterns of *Staphylococcus* spp. isolates from canine specimens submitted to a diagnostic laboratory in Tennessee, USA (2006–2017).

	**Percent of isolates (number resistant/total number of isolates)**			
	***S. pseudintermedius***	***S. aureus***	***S. coagulans***	***S. schleiferi***
Aminoglycosides	19.8 (1270/6413)	4.6 (15/326)	27.8 (238/857)	32.1 (51/159)
*β*-lactams	84.4 (5444/6453)	91.5 (302/330)	44.2 (380/860)	66.7 (108/162)
Fluoroquinolones	29.5 (1893/6422)	33.4 (109/326)	38.7 (331/856)	49.7 (80/161)
Folate inhibitors	44.4 (2863/6447)	4.2 (14/330)	1.2 (10/859)	2.5 (4/162)
Lincosamides	39.1 (2517/6438)	35.4 (116/328)	3.4 (29/858)	8.6 (14/162)
Macrolides	38.8 (2498/6439)	47.3 (156/330)	3.1 (27/860)	8.6 (14/162)
Phenicols	5.4 (345/6448)	2.4 (8/330)	0 (0/859)	0.6 (1/162)
Tetracyclines	48.0 (3085/6424)	6.7 (22/329)	2.4 (21/859)	6.8 (11/162)
AMR^a^	86.7 (5596/6453)	92.7 (306/330)	57.0 (490/860)	77.2 (125/162)
MDR^b^	45.5 (2933/6453)	40.9 (135/330)	20.0 (172/860)	27.2 (44/162)
MR^c^	30.8 (1908/6195)	37.4 (117/313)	32.2 (261/811)	56.9 (87/153)

**Notes.**

a Antimicrobial resistant.

b Multidrug resistant.

c Methicillin resistant.

Light gray shading indicates antimicrobial resistance (resistance to one or more antimicrobial classes). Dark gray shading indicates multidrug resistance (resistance to three or more antimicrobial classes) or methicillin resistance.

**Table 3:** Most common patterns of antimicrobial resistance among multidrug and methicillin resistant *Staphylococcus* spp. isolated from canine specimens submitted to a diagnostic laboratory in Tennessee, USA (2006–2017).

**Antimicrobial resistance pattern**		
***Multidrug resistant isolates***	**Percent of multidrug- resistant isolates**	**Number of multidrug- resistant isolates**
Aminoglycosides, *β*-lactams, fluoroquinolones, tetracyclines, folate pathway inhibitors, lincosamides, macrolides	23.9	784
*β*-lactams, fluoroquinolones, tetracyclines, folate pathway inhibitors, lincosamides, macrolides	20.5	672
*β*-lactams, tetracyclines, folate pathway inhibitors	7.1	232
Aminoglycosides, *β*-lactams, fluoroquinolones	4.9	162
*β*-lactams, tetracyclines, folate pathway inhibitors, lincosamides, macrolides	4.7	154
***Methicillin resistant isolates***	**Percent of methicillin- resistant isolates**	**Number of methicillin- resistant isolates**
Aminoglycosides, *β*-lactams, fluoroquinolones, tetracyclines, folate pathway inhibitors, lincosamides, macrolides	28.3	672
*β*-lactams, fluoroquinolones, tetracyclines, folate pathway inhibitors, lincosamides, macrolides	21.4	508
*β*-lactams	6.1	145
Aminoglycosides, *β*-lactams, fluoroquinolones	4.8	115
*β*-lactams, fluoroquinolones	4.2	100

Both multidrug and methicillin resistance were more common among *Staphylococcus* spp. isolates from hospital patient specimens compared to those from referral submissions (Table 4). Among isolates from hospital patient specimens, 46.9% were multidrug-resistant, and 34.9% exhibited methicillin resistance. Isolates from skeletal specimens had the highest levels of MDR (51.3%) and methicillin resistance (43.6%), followed by cutaneous isolates (45.8% MDR, 37.1% methicillin-resistant) (Table 4).

**Table 4:** Multidrug and methicillin resistance by clinical characteristics among *Staphylococcus* spp. isolated from canine specimens submitted to a diagnostic laboratory in Tennessee, USA (2006–2017).

**Characteristic**	**Multidrug resistance**			**Methicillin resistance**		
	**Number**	**Percent**	**95% CI** ^a^	**Number**	**Percent**	**95% CI** ^a^
Specimen site						
Skin	2011/4387	45.8	44.4, 47.3	1552/4182	37.1	35.7, 38.6
Skeletal	236/460	51.3	46.7, 55.9	186/427	43.6	38.9, 48.3
Mucosa	49/122	40.2	31.7, 49.0	26/113	23.0	15.9, 31.3
Urine/bladder	350/1041	33.6	30.8, 36.5	181/1004	18.0	15.7, 20.5
Ear	432/1310	33.0	30.5, 35.6	303/1274	23.8	21.5, 26.2
Other	205/484	42.4	38.0, 46.8	124/471	26.3	22.5, 30.4
Patient type						
Hospital	1476/3149	46.9	45.1, 48.6	1040/2979	34.9	33.2, 36.6
Referral	1779/4556	39.1	37.6, 40.5	1292/4393	29.4	28.1, 30.8

**Notes.**

a Confidence interval.

### Determinants of MDR and methicillin resistance

#### (a) MDR model

**Figure 1:**
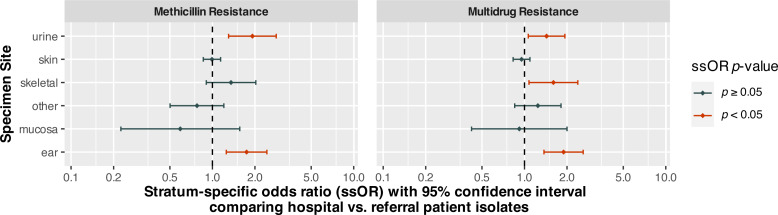
Stratum-specific odds ratios comparing canine *Staphylococcus* spp. isolates from hospital and referral patients by specimen site, from submissions to a diagnostic laboratory in Tennessee, USA (2006–2017).

Specimen site, patient type, and organism species were statistically significant (*p* < 0.0001) predictors of MDR (Table 5). There was also a significant (*p* = 0.0004) interaction between specimen site and patient type. Holding patient type and specimen site constant, *S. coagulans* (OR = 0.26, 95% CI [0.21–0.33], *p* < 0.0001) and *S. schleiferi* (OR = 0.30, 95% CI [0.19–0.48], *p* < 0.0001) isolates had significantly lower odds of multidrug resistance compared to *S. pseudintermedius*. On the other hand, the odds of multidrug resistance among *S. aureus* isolates were not significantly different from those of *S. pseudintermedius* (*p* = 0.1695). Isolates from hospital patients had significantly (*p* < 0.0001) higher odds of MDR than those from referral submissions. However, the interaction between specimen site and patient type indicated that the odds ratios comparing these groups differed depending upon specimen site. Stratum-specific odds ratios comparing hospital and referral patients for each specimen site are displayed in Fig. 1. The odds of MDR for isolates from hospital patient specimens were significantly higher than referral submissions for urine/bladder (OR = 1.43, 95% CI [1.06–1.93], *p* = 0.0178), skeletal (OR = 1.60, 95% CI [1.08–2.39], *p* = 0.0203), and ear (OR = 1.89, 95% CI [1.37–2.60], *p* < 0.0001) specimens.

**Table 5:** Significant predictors of multidrug and methicillin resistance of *Staphylococcus* spp. isolated from canine specimens submitted to a diagnostic laboratory in Tennessee, USA (2006–2017).

**Parameter**	**Classification**	**Multidrug resistance**		**Methicillin resistance**	
		*β* ^a^ **(95% CI^b^)**	***p*-value**	*β* ^a^ **(95% CI^b^)**	***p*-value**
*Main effects*					
Intercept		**−0.64 (−0.80, −0.49)** ^*^	<0.0001	**−1.44 (−1.61, −1.26)** ^*^	<0.0001
Specimen site			<0.0001		<0.0001
	Skin	**0.54 (0.37, 0.71)** ^*^	<0.0001	**0.78 (0.58, 0.97)** ^*^	<0.0001
	Skeletal	0.41 (0.07, 0.76)^*^	0.0186	**0.84 (0.48, 1.19)** ^*^	<0.0001
	Mucosa	0.38 (−0.19, 0.96)	0.1906	0.47 (−0.18, 1.11)	0.1581
	Urine/bladder	**−0.34 (−0.59, −0.10)** ^*^	0.0057	**−0.62 (−0.94, −0.30)** ^*^	0.0001
	Other	0.18 (−0.16, 0.51)	0.3016	**0.45 (0.07, 0.82)** ^*^	0.0187
	Ear	Ref.	–	Ref.	–
Patient type					
	Hospital	0.64 (0.32, 0.96)^*^	<0.0001	**0.56 (0.23, 0.89)** ^*^	0.0010
	Referral	Ref.	–	Ref.	–
Species of organism			<0.0001		0.0002
	*S. aureus*	−0.19 (−0.46, 0.08)	0.1695	0.17 (−0.14, 0.47)	0.2882
	*S. coagulans*	**−1.33 (−1.56, −1.11)** ^*^	<0.0001	0.07 (−0.14, 0.28)	0.4892
	*S. schleiferi*	**−1.20 (−1.65, −0.74)** ^*^	<0.0001	**0.98 (0.58, 1.38)** ^*^	<0.0001
	*S. pseudintermedius*	Ref.	–	Ref.	–
*Interaction term*					
Patient type	Specimen site		0.0004		0.0007
Hospital	Skin	**−0.68 (−1.02, −0.35)** ^*^	<0.0001	**−0.56 (−0.91, −0.21)** ^*^	0.0016
	Skeletal	−0.17 (−0.68, 0.35)	0.5259	−0.25 (−0.77, 0.27)	0.3381
	Mucosa	−0.72 (−1.57, 0.12)	0.0947	**−1.08 (−2.11, −0.05)** ^*^	0.0396
	Urine/bladder	−0.28 (−0.71, 0.16)	0.2133	0.09 (−0.42, 0.60)	0.7155
	Other	−0.42 (−0.92, 0.08)	0.0985	**−0.81 (−1.36, −0.25)** ^*^	0.0042
Referral	Ear	Ref.	–	Ref.	–

**Notes.**

a Coefficient estimate.

b Confidence interval.

* Bold values indicate significance at *p* < 0.05.

**Figure 2:**
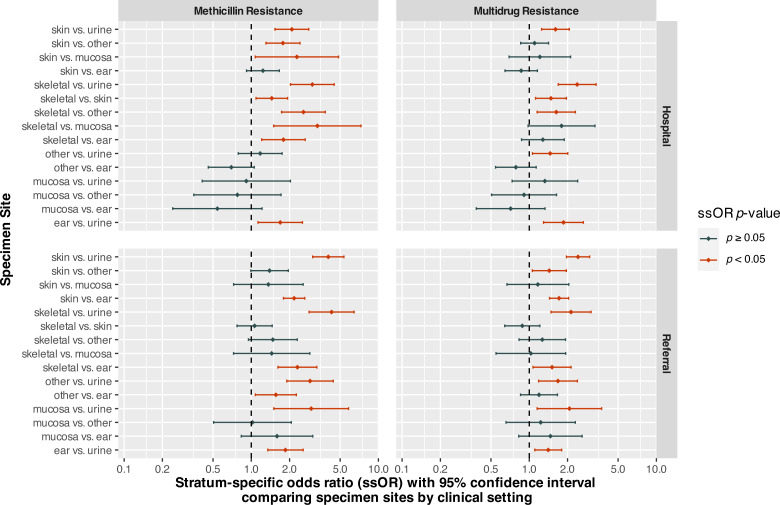
Stratum-specific odds ratios comparing specimen sites by clinical setting for *Staphylococcus* spp. isolates from canine specimens submitted to a diagnostic laboratory in Tennessee, USA (2006–2017).

Stratum-specific odds ratios comparing different specimen sites, while controlling for clinical setting, are displayed in Fig. 2. For isolates from hospital patients, odds of MDR were significantly higher for skeletal isolates compared to those from urine/bladder (OR = 2.38, 95% CI [1.69–3.36], *p* < 0.0001), skin (OR = 1.48, 95% CI [1.12–1.96], *p* = 0.0058), and other (OR = 1.63, 95% CI [1.15–2.31], *p* = 0.0056) sites (Fig. 2). In addition, hospital patient isolates from skin (OR = 1.61, 95% CI [1.25–2.07], *p* = 0.0002), ear (OR = 1.86, 95% CI [1.30–2.67], *p* = 0.0008), and other (OR = 1.46, 95% CI [1.06–2.01], *p* = 0.0204) sites had higher odds of MDR compared to urine/bladder isolates.

For isolates from referral submissions, skeletal specimens also had higher odds of MDR compared to urine/bladder (OR = 2.13, 95% CI [1.48–3.06], *p* < 0.0001) and ear (OR = 1.51, 95% CI [1.07–2.13], *p* = 0.0186) specimens. Skin specimens had significantly higher odds of MDR compared to those from ear (OR = 1.72, 95% CI [1.44–2.04], *p* < 0.0001), urine/bladder (OR = 2.42, 95% CI [1.96–2.98], *p* < 0.0001), and other (OR = 1.44, 95% CI [1.06–1.96], *p* = 0.0207) sites. The odds of MDR for isolates from ear (OR = 1.41, 95% CI [1.11–1.80], *p* = 0.0057), mucosal (OR = 2.07, 95% CI [1.15–3.71], *p* = 0.0146), and other (OR = 1.68, 95% CI [1.18–2.39], *p* = 0.0037) sites were significantly higher than those from urine/bladder isolates.

#### (b) Methicillin resistance model

As was the case for the MDR model, species of organism (*p* = 0.0002), specimen site (*p* < 0.0001) and patient type (*p* = 0.0010) were all significant predictors of methicillin resistance (Table 5). There was also a significant interaction (*p* = 0.0007) between specimen site and patient type in the methicillin model. *S. schleiferi* (OR = 2.66, 95% CI [1.78–3.98], *p* < 0.0001) isolates had higher odds of methicillin resistance when compared to *S. pseudintermedius*, while *S. aureus* and *S. coagulans* did not. As in the MDR model, the interaction term indicated that the odds of methicillin resistance for hospital patient isolates compared to those of isolates from referral submissions differed based on specimen site. While the odds of methicillin resistance for isolates from hospital patients were significantly higher than those from referral submissions for urine/bladder (OR = 1.92, 95% CI [1.30–2.83], *p* = 0.0010) and ear (OR = 1.75, 95% CI [1.25–2.43], *p* = 0.0010) specimens, they did not differ significantly for isolates from any other specimen site (Fig. 1).

Among hospital patient specimens, the odds of methicillin resistance for skeletal isolates were significantly higher than those from urine/bladder (OR = 3.02, 95% CI [2.03–4.49], *p* < 0.0001), skin (OR = 1.45, 95% CI [1.09–1.93], *p* = 0.0110), mucosal (OR = 3.31, 95% CI [1.50–7.29], *p* = 0.0030), ear (OR = 1.79, 95% CI [1.21–2.65], *p* = 0.0038), and other (OR = 2.57, 95% CI [1.73–3.82], *p* < 0.0001) sites (Fig. 2). Skin isolates had significantly higher odds of methicillin resistance compared to those from urine/bladder (OR = 2.09, 95% CI [1.54–2.83], *p* < 0.0001), mucosal (OR = 2.28, 95% CI [1.08–4.85], *p* = 0.0316), and other (OR = 1.77, 95% CI [1.30–2.42], *p* = 0.0003) sites.

Among referral submissions, the odds of methicillin resistance did not differ significantly between skeletal and skin (*p* = 0.7110), mucosal (*p* = 0.2947), or other (*p* = 0.0837) isolates. However, the odds of methicillin resistance among isolates from skeletal specimens were significantly greater than those of urine/bladder (OR = 4.28, 95% CI [2.85–6.44], *p* < 0.0001) and ear (OR = 2.31, 95% CI [1.62–3.28], *p* < 0.0001) specimens (Fig. 2). Skin isolates had higher odds of methicillin resistance than isolates from urine/bladder (OR = 4.03, 95% CI [3.04–5.35], *p* < 0.0001) and otic (OR = 2.17, 95% CI [1.79–2.63], *p* < 0.0001) sites, but did not differ significantly from mucosal (*p* = 0.3356) or other (*p* = 0.0560) isolates. In addition, referral patient isolates from the ear (OR = 1.86, 95% CI [1.35–2.56], *p* = 0.0001), mucosal (OR = 2.96, 95% CI [1.50–5.82], *p* = 0.0017), and other (OR = 2.90, 95% CI [1.90–4.42], *p* < 0.0001) sites had greater odds of methicillin resistance compared to urine/bladder isolates.

## Discussion

This study investigated predictors of multidrug resistance and methicillin resistance among *Staphylococcus* spp. isolated from canine clinical specimens submitted to the Clinical Bacteriology and Mycology Laboratory at the University of Tennessee College of Veterinary Medicine between 2006 and 2017. Significant differences in the odds of MDR and methicillin resistance were identified between staphylococcal species, with the highest odds of MDR being observed among *S. pseudintermedius* and *S. aureus*, while *S. schleiferi* isolates had the highest odds of methicillin resistance. These findings are consistent with those from previous research conducted at UTCVM, which reported that *S. coagulans* and *S. schleiferi* had relatively high levels of oxacillin resistance compared to *S. pseudintermedius* and *S. aureus*, but generally exhibited lower levels of resistance to non-*β*-lactam antibiotics (Jones et al., 2007). Similarly, a study from the University of Pennsylvania reported that susceptibility to most non-*β*-lactam antimicrobials tended to be high among *S. coagulans* and *S. schleiferi* isolates, despite substantial levels of oxacillin resistance among these organisms (48% and 62%, respectively) (Cain, Morris & Rankin, 2011). The relatively high level of methicillin resistance observed among *S. schleiferi* isolates in this and previous studies is clinically relevant given the increasing recognition of this organism as an opportunistic pathogen in canine otitis and pyoderma, particularly in recurrent infections (Frank et al., 2003; May, Kinyon & Noxon, 2012; Lee et al., 2019). However, it is important to note that while *S. pseudintermedius* had comparatively lower odds of methicillin resistance, isolation of this organism was far more common than *S. schleiferi*, and nearly one-third were MRSP (30.8%). In addition, the vast majority of MRSP were also multidrug-resistant (90.5%), which is concerning because few treatment options are available for infections with multidrug- and methicillin-resistant isolates. Also of note was the substantial level of MRSA (37.4%) among *S. aureus* isolates in the current study, the majority of which were also multidrug-resistant (78.6%). Despite the relatively small fraction of isolates in the study represented by the 92 MDR-MRSA isolates, this is an important finding with respect to human health risk since *S. aureus* is primarily a commensal and opportunistic pathogen of humans (Tong et al., 2015). While transmission of *S. aureus* from humans to companion animals is believed to occur more frequently than the reverse, household pets may play an important role in *S. aureus* transmission dynamics (Mork et al., 2020). Thus, the identification of MRSA and MDR-MRSA isolates in this study highlights the importance of cooperation between the human and veterinary medical communities to address human and animal health risks.

Significant differences in the odds of MDR and methicillin resistance were also observed with respect to the clinical setting. “Hospital” specimens were obtained from patients of the UTCVM Veterinary Medical Center, which is a tertiary referral hospital, while “referral” specimens were those processed at the Bacteriology Laboratory that were collected from patients of outside clinics, including primary care practices, in the surrounding region. Thus, the identification of differences between isolates from hospital and referral specimens was not surprising, since patients from these settings are likely to differ with respect to risk factors for MDR and methicillin resistance such as frequency of recent veterinary visits (Lehner et al., 2014; Grönthal et al., 2015), history of hospitalization (Lehner et al., 2014), concomitant diseases (Ortiz-Díez et al., 2020), antimicrobial use (Faires et al., 2010; Soares Magalhães et al., 2010; Cain, Morris & Rankin, 2011; Huerta et al., 2011; Weese et al., 2012; Eckholm et al., 2013; Grönthal et al., 2015; Zur, Gurevich & Elad, 2016; Ortiz-Díez et al., 2020), and use of immunosuppressive medications (Lehner et al., 2014; Ortiz-Díez et al., 2020). Interestingly, however, these differences did not persist across all specimen sites, and were only observed among isolates from urine/bladder (MDR and methicillin resistance), ear (MDR and methicillin resistance), and skeletal (MDR only) specimens. This could potentially reflect differences in treatment practices, including diagnostic testing and antimicrobial use practices, with respect to body site or system.

For instance, the odds of MDR and methicillin resistance among otic/ear isolates from hospital patients were higher than those from referral submissions. However, no such difference was observed for cutaneous isolates, consistent with a previous study that reported staphylococci isolated from canine pyoderma in teaching hospital and primary care settings had similar odds of methicillin resistance (Eckholm et al., 2013). This was an interesting finding given that pyoderma and otitis, particularly recurrent infections, are both often secondary to underlying allergic diseases such as atopic dermatitis or food allergy (Rosser, 2004; Loeffler & Lloyd, 2018). Symptoms of allergic disease in dogs are some of the most common presenting complaints in companion animal practice, and chronic allergic conditions are frequently managed in the primary care setting. The finding that cutaneous isolates from different clinical settings had comparable odds of MDR and methicillin resistance may suggest that patients with skin infections caused by staphylococci tend to have a history of risk factors such as systemic antimicrobial use regardless of whether they are being treated by their primary veterinarian or have been referred to a specialty service. On the other hand, treatment of canine otitis externa is typically guided by cytology and involves targeted treatment with topical formulations that include non-*β*-lactam antibiotics. The findings of this study suggest that companion animal veterinarians in the region may encounter antimicrobial treatment failures in cases of canine pyoderma, particularly in dogs with risk factors for recurrent infections.

This study also found that urine/bladder isolates from referral patient specimens tended to have significantly lower odds of MDR and methicillin resistance compared to those from hospital patients, and that isolates from urine/bladder specimens tended to have lower odds of MDR and methicillin resistance than most other sites. These findings may also reflect typical treatment practices and patients’ history of risk factors for antimicrobial resistance. For instance, diagnostic testing tends to be performed relatively early in the course of urinary tract disease when bacterial etiology is suspected. Clinical signs consistent with cystitis are another common presenting complaint for dogs in the primary care setting, and in-house urinalysis is typically part of the minimum database of diagnostics performed prior to treatment with systemic antimicrobials. In addition, bacterial culture and susceptibility testing are recommended whenever feasible, including for first-time onset of clinical signs (Weese et al., 2021). The observed difference between hospital and referral specimens could also suggest that dogs with bacterial urinary tract disease that does not respond to antimicrobial treatment tend to be referred to specialty practice for further workup relatively early in the course of illness. This is unsurprising, because relapse and recurrence of bacterial urinary tract disease in dogs are typically indicative of an underlying issue such as endocrinopathy, renal disease, urolithiasis, conformational abnormalities or neoplasia, among others (Weese et al., 2021).

Skeletal (joint and bone) isolates had considerable levels of MDR and methicillin resistance in this study, and tended to have higher odds of these outcomes compared to other body sites, particularly in the hospital setting. A substantial percentage (43.6%) of *Staphylococcus* spp. isolates from joint and bone specimens were methicillin-resistant, and over half (51.3%) were multidrug-resistant. This finding could be indicative of high levels of MDR and methicillin resistance in post-operative *Staphylococcus* spp. infections following orthopedic surgery, a concern that has been raised by other researchers and which is worrisome because treatment of these infections may be challenging (Weese et al., 2012). Among canine *S. aureus* isolates, a previous study conducted in the United Kingdom identified surgical implants as a risk factor for methicillin resistance (Soares Magalhães et al., 2010). Further investigation of patient medical history is warranted to identify specific drivers of the observed levels of MDR and methicillin resistance among skeletal isolates in this population.

### Strengths and limitations

Detailed medical history, including antimicrobial use, other medications, concomitant diseases, surgical procedures, number of veterinary visits, and recent hospitalization, which have previously been identified as risk factors for MDR and/or methicillin resistance among canine *Staphylococcus* spp. isolates (Faires et al., 2010; Soares Magalhães et al., 2010; Cain, Morris & Rankin, 2011; Huerta et al., 2011; Weese et al., 2012; Eckholm et al., 2013; Lehner et al., 2014; Grönthal et al., 2015; Zur, Gurevich & Elad, 2016; Ortiz-Díez et al., 2020), were not available for analysis in the current study. In addition, patient signalment information was missing for a large portion of referral submissions, and was therefore excluded from the analysis. Finally, this study focused on four clinically relevant *Staphylococcus* species and, other than *S. schleiferi,* did not include coagulase-negative staphylococci (CoNS), which have historically been considered non-pathogenic but may be important reservoirs for antimicrobial resistance genes (Becker, Heilmann & Peters, 2014; Teixeira, De Oliveira Ferreira & De Araújo Penna, 2019). Given the increasing recognition of CoNS and their role in drug-resistant infections in humans (Becker, Heilmann & Peters, 2014), future studies investigating antimicrobial resistance patterns in canine CoNS may be warranted.

Despite the above limitations, the present study provides useful information to guide treatment decisions for veterinary practitioners. Moreover, the large sample size allowed for high statistical power to detect differences between sub-populations, and therefore provides important information about *Staphylococcus* spp. organisms that are isolated relatively infrequently in comparison to *S. pseudintermedius*. Identifying sites of infection where *Staphylococcus* spp. isolates are more likely to exhibit multidrug or methicillin resistance may also be useful for veterinary practitioners because it may influence clinicians’ decisions regarding when to pursue diagnostic testing, as well as antimicrobial selection when empirical treatment is necessary.

## Conclusions

*Staphylococcus* spp. isolates had substantial levels of multidrug resistance and methicillin resistance. Moreover, the methicillin-resistant isolates were frequently multidrug-resistant, particularly among *S. pseudintermedius* and *S. aureus*. The finding that the most common pattern of resistance among methicillin-resistant *Staphylococcus* isolates included resistance to seven of the eight classes of antimicrobials assessed in this study highlights the treatment challenge these organisms pose in companion animal practice. The high proportion of skeletal isolates that exhibited multidrug resistance and methicillin resistance is especially concerning.

The fact that the odds of MDR and methicillin resistance for referral patient isolates from some sites were comparable to those of hospital patients implies that, regardless of clinical setting, judicious antimicrobial use practices are essential to limit selection pressure and slow development of AMR. To decrease the likelihood of treatment failures when bacterial infection is suspected, antimicrobial treatment should be guided by diagnostics such as cytology, bacterial culture and susceptibility testing whenever possible. Surveillance of antimicrobial resistance trends at the clinic level may also be warranted, particularly if treatment failures are repeatedly documented. In light of the close contact between companion animals and their owners, limiting further increases of antimicrobial resistance among canine staphylococci is an important goal from both a veterinary and human public health perspective. The findings of this study contribute to the evidence base supporting One Health approaches to curb the threats of multidrug and methicillin resistance, particularly among staphylococci with zoonotic potential.

##  Supplemental Information

10.7717/peerj.15012/supp-1 Supplemental Information 1 Study Data

## List of abbreviations

AMR: antimicrobial resistance
CI: confidence interval
CoNS: coagulase-negative staphylococci
CLSI: Clinical and Laboratory Standards Institute
GEE: generalized estimating equations
IACUC: Institutional Animal Care and Use Committee
IQR: interquartile range
MDR: multidrug resistance
MIC: minimum inhibitory concentration
MRSA: methicillin-resistant *Staphylococcus aureus*
MRSP: methicillin-resistant *Staphylococcus pseudintermedius*
OR: odds ratio
PCR: polymerase chain reaction
QIC_u_: modified quasi-likelihood information criterion
SIG: *Staphylococcus intermedius* group
TMS: trimethoprim/sulfamethoxazole
UTCVM: University of Tennessee College of Veterinary Medicine

## Additional Information and Declarations

### Data Availability

The following information was supplied regarding data availability: The raw data is available in the Supplemental File.
